# An Account of the Late Epidemic of Scarlatina in Newcastle and Its Neighbourhood

**Published:** 1847-07

**Authors:** 


Art. VIII.—*An Account of the late Epidemic of Scarlatina in Newcastle and its Neighbourhood.* By Edward Charlton, m.d. Edin., &c.—1847. Pamphlet, 8vo, pp. 62.

Dr. Charlton has laudably assumed the office of historian for Newcastle, in so far as relates to the epidemic of scarlatina, which prevailed more extensively in that town during the summer and autumn of 1846, than at any period since 1778, when Dr. John Clark recorded its progress and characteristics. The well-written tract before us professes to contain little of novelty, and adds rather to our experience than our science. Its principal defect is the absence of numerical data, and of a more minute comparison of the relations which the origin and progress of the epidemic bore to the meteorological phenomena. General statements are becoming less and less admissible in medical writings. There are, however, some interesting facts detailed, and it is probable that Dr. Charlton has made the best use he could of the materials at his disposal. In turning to the head of "Cause," we do not find a word as to the influence of famine; other more efficient febrific agents are, however, noticed.

"The fever was not confined to the ancient and low-lying streets along the water-side on either bank of the Tyne, it spread through the streets and courts higher above the level of that river, especially to those recently built houses inhabited by numerous families, about Arthur's Hill, to the densely-crowded courts and narrow lanes at the foot of Westgate street, while in Gateshead its ravages were greatest in the wretched purlieus of Leonard's court, and the cottages about Busy Burn." (pp. 24-5.)

Arthur's Hill, and Leonard's Court, so distinguished for scarlatina, are in an Irish condition. Impassable quagmires, unpaved streets, "four or five beds in one small apartment, lighted by a single window which is rarely opened;" such is a picture corresponding very graphically to the localities of fever in Ireland. It is clear that while we think of the poor man's stomach, we must not forget his lungs.

